# Radiologic Evaluation of Portosystemic Shunts in Humans and Small Animals: Review of the Literature with Clinical Case Reports

**DOI:** 10.3390/diagnostics13030482

**Published:** 2023-01-28

**Authors:** Nejc Umek, Domen Plut, Martina Krofič Žel, Aleksandra Domanjko Petrič

**Affiliations:** 1 Institute of Anatomy, Faculty of Medicine, University of Ljubljana, Korytkova ulica 2, 1000 Ljubljana, Slovenia; 2 Department of Radiology, Faculty of Medicine, University of Ljubljana, Vrazov trg 2, 1000 Ljubljana, Slovenia; 3 Clinical Institute of Radiology, University Medical Centre Ljubljana, Zaloška cesta 7, 1000 Ljubljana, Slovenia; 4 Small Animal Clinic, Veterinary Faculty, University of Ljubljana, Gerbičeva 60, 1000 Ljubljana, Slovenia

**Keywords:** portosystemic shunt, ultrasonography, computational tomographic angiography, anatomy, humans, dogs, cats

## Abstract

The portal venous system is a network of vessels that carry blood from the capillary beds of the major abdominal organs to the liver. During embryology, the portal venous system can develop aberrantly, leading to vascular connections between the portal and systemic venous circulation known as portosystemic shunts. The purpose of this comparative review with a few short representative case reports was to present the similarities and differences in portosystemic shunts in humans and small animals and their radiologic evaluation. Aberrant vascular connections between the portal and systemic venous circulation enable portal blood to bypass metabolism and detoxification in the liver, leading to significant clinical implications. Portosystemic shunts are very rare in humans, but these connections are much more common in small animals, affecting up to 0.6% of small animals. Portosystemic shunts can be congenital or acquired and are divided into intrahepatic and extrahepatic types. Because of its ability to accurately assess abdominal structures, large vessels, and their flow dynamics without anesthesia, ultrasonography has become the first imaging modality employed for the diagnostic evaluation of portosystemic shunts in both humans and small animals. This is usually followed by contrast-enhanced computed tomographic angiography in order to better define the exact shunt anatomy and to plan treatment. It is important to understand the embryology, anatomy, pathology, and pathophysiology of portosystemic shunts in order to understand the findings of radiologic imaging and to initiate appropriate treatment.

## 1. Introduction

The portal venous system is a network of vessels that drains blood from the capillary beds of most of the abdominal organs (i.e., gastrointestinal tract, pancreas, spleen) to the liver. The nutrients and other blood constituents, such as microbes and toxins, are metabolised and detoxified by the hepatocytes and are subsequently delivered back into the systemic circulation through the hepatic veins [1]. Aberrant vascular communications between the portal and systemic venous circulation (portosystemic shunts) allow portal blood to bypass metabolism and detoxification in the liver and lead to significant clinical ramifications, which mainly depend on the ratio of blood flow through the shunt. Therefore, the clinical picture of the shunt can range from an asymptomatic patient with incidentally detected elevated liver enzymes or diagnostic imaging findings to a symptomatic patient with severe symptoms, such as encephalopathy that leads to neurodevelopmental delay, hepatopulmonary syndrome, portopulmonary hypertension, and regenerative liver nodules. The estimated incidence of portosystemic shunts is from 1/30,000 to 1/50,000 live births in humans; however, in small animals, these communications are much more prevalent, affecting up to 6/1000 small animals [2]. Rapidly evolving advancements in imaging modalities allow for the increased recognition of congenital or acquired portosystemic shunts in humans and animals [3,4]. The aim of this paper was to a present a thorough overview of the embryology, anatomy, and radiologic imaging of portosystemic shunts in humans and small animals (i.e., dogs and cats).

The purpose of this review, with some representative cases, was to present the similarities and differences in anatomical defects between humans and small animals, which could contribute to the understanding of comparative mammalian embryology. It provides an overview of the developmental abnormalities of the portosystemic vasculature and may help veterinaries, clinicians working with laboratory animals, and clinicians who are not familiar with these states to better understand the pathology and radiologic evaluation of portosystemic shunts. A few representative case reports, in addition to the extensive literature review, are discussed for illustrative purposes. Two human and three veterinary cases of congenital intra- and extrahepatic portosystemic shunts are discussed. The veterinary cases were selected because they represent the extrahepatic portosystemic shunts that are different in origin, whereas human cases were selected because they represent one intrahepatic and one extrahepatic shunt.

## 2. Clinical Case Presentations

### 2.1. Case A (Human)

**Figure 1:**
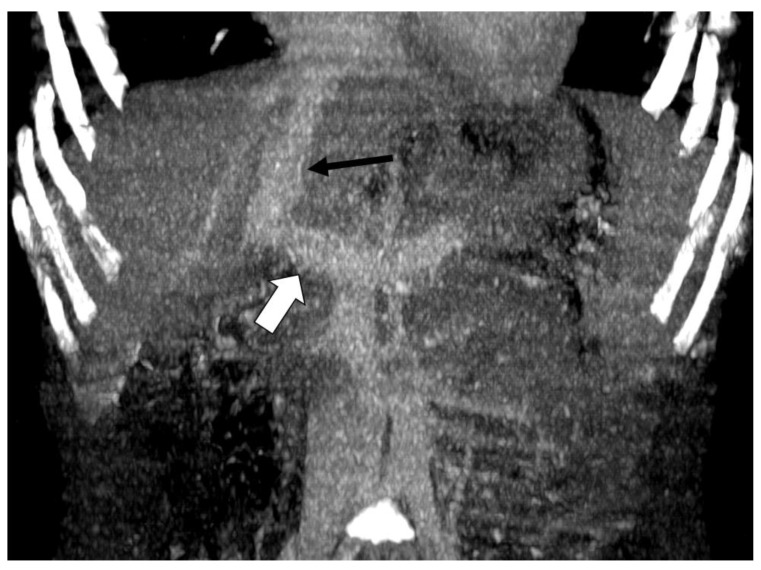
Coronal contrast-enhanced computed tomography image of the abdominal vessels in an 8-year-old boy. The white arrow represents a direct vascular connection between the portal vein and the dilated hepatic vein (black arrow).

An 8-year-old boy was admitted to the University Medical Centre Ljubljana because of severe abdominal pain and recurrent melena. Doppler ultrasonography showed an aberrant intrahepatic tubular vessel. Contrast-enhanced computed tomography was performed and showed a direct vascular connection between the portal vein (white arrow) and the dilated left hepatic vein—an intrahepatic portosystemic venous shunt (black arrow) (Figure 1) [5]. The right portal vein had a small diameter, suggesting that the majority of the portal blood flows through the left side, bypassing the liver. The treatment for this intrahepatic portosystemic venous shunt was embolization of the shunt. The boy has been doing well since this procedure.

### 2.2. Case B (Human)

**Figure 2:**
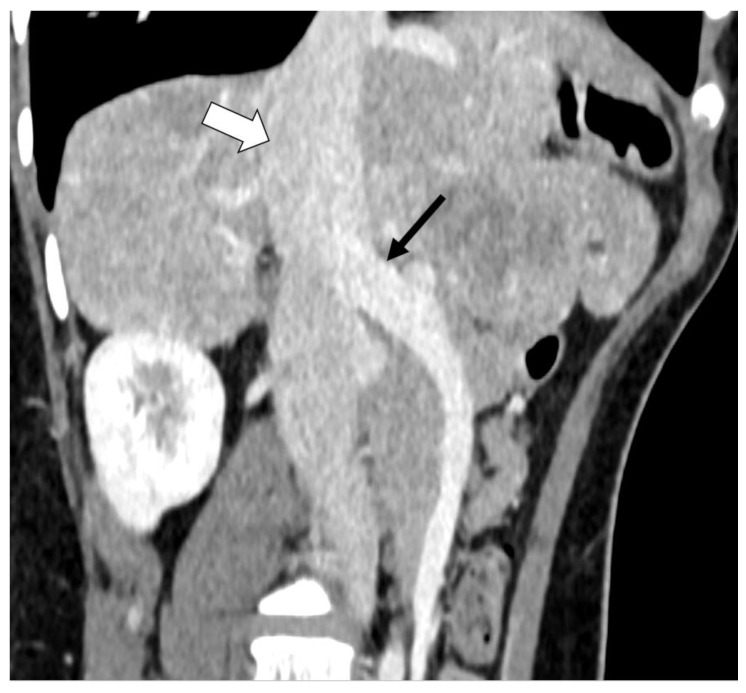
Contrast-enhanced computed tomography image of the abdominal vessels in a 14-year-old girl. The black arrow represents the short portal vein connected directly to the wide inferior vena cava (white arrow).

Abnormal liver function tests were noted during the routine examination of a 14-year-old girl. Abdominal ultrasonography revealed a large mass in the right lobe of the liver and a large mass of similar structure in the epigastrium. The portal vein was not identified. Contrast-enhanced computed tomography showed a short main portal vein (black arrow) connected directly to a wide inferior vena cava (white arrow) (Figure 2), consistent with a type 1 extrahepatic portosystemic venous shunt, also called Abernethy [6,7]. In this type of shunt, the majority of the portal blood bypasses the liver. The girl developed typical symptoms with large benign liver lesions and changes in the brain on MRI that were suggestive of hepatic encephalopathy. The only treatment for this malformation is a liver transplant, which the girl recently received.

### 2.3. Case C (Dog)

**Figure 3:**
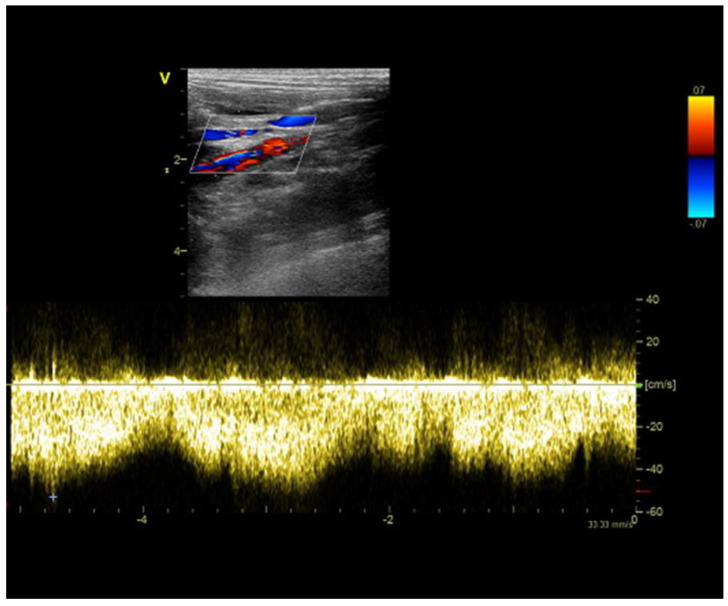
Ultrasound image of an anomalous vessel with high blood flow velocity medial to the right kidney in a 1-year-and-8-month-old female Yorkshire terrier dog.

**Figure 4:**
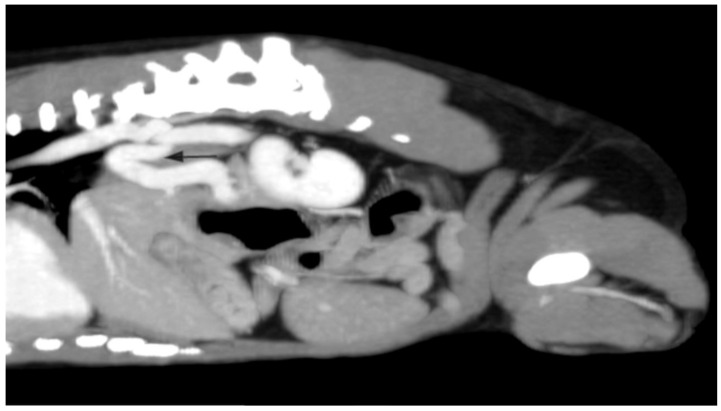
Sagittal contrast-enhanced computed tomography image of a tortious shunting vessel (arrow), originating from the left gastric vein and joining the azygos vein in a 1-year-and-8-month-old female Yorkshire terrier dog.

A 1-year-and-8-month-old female Yorkshire terrier dog was admitted to the Small Animal Clinic of Veterinary Faculty in Ljubljana due to episodes of weakness that improved after therapy with metronidazole, lactulose, and a low protein diet. An abdominal ultrasound showed a small liver, hypoperfusion of the intrahepatic portal branches, and a distended gallbladder. Medial to the right kidney, an anomalous vessel with high blood flow velocity (50–94 cm s^−1^) was noted (Figure 3). The contrast-enhanced computed tomography revealed a tortuous shunting vessel originating from the left gastric vein and joining the azygos vein, a left gastroazygous shunt (Figure 4), which is the most common subtype of the portoazygos shunt [8]. The shunting vessel was surgically closed with cellophane banding.

Extrahepatic shunts are common in toy and small breed dogs, and the signalment in the presented clinical case is typical [9]. In addition, abdominal ultrasonography revealed an anomalous vessel medial to the right kidney. According to the literature, in the presence of a portoazygos shunt, the azygos vein, which is not normally visible, is prominent in this region [10].

### 2.4. Case D (Dog)

A 1-year-and-7-month-old mixed breed female dog presented with a history of weakness episodes lasting from a few hours to 2 days, an epileptoid seizure after vaccination, and abnormal liver function tests. The signalment in this clinical case was atypical, as it was an adult mixed breed dog. More frequently, congenital portosystemic shunts are found in young purebred dogs [8].

**Figure 5:**
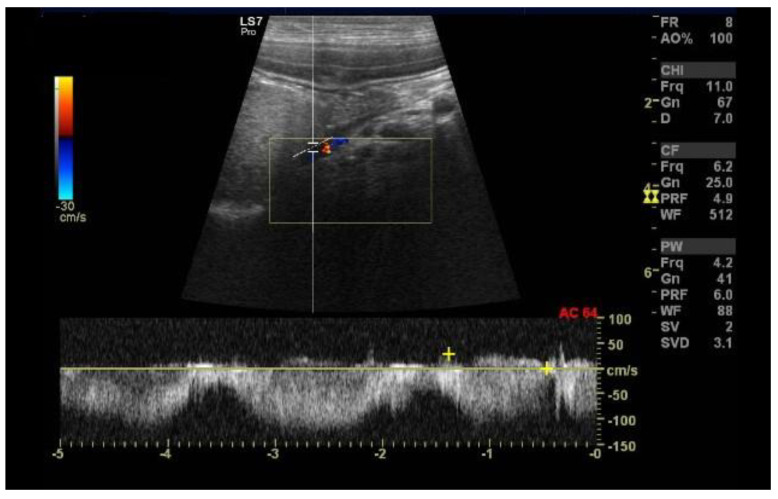
Ultrasound image of an anomalous shunting vessel visible from the right side in a 1-year-and-7-month-old mixed breed female dog.

An abdominal ultrasound revealed a small liver with hypoperfusion of the intrahepatic portal branches and sediment in the urinary bladder (Figure 5). Urinary sediment and uroliths result from renal ammonium urate excretion and are a common finding in patients with portosystemic shunts [11].

**Figure 6:**
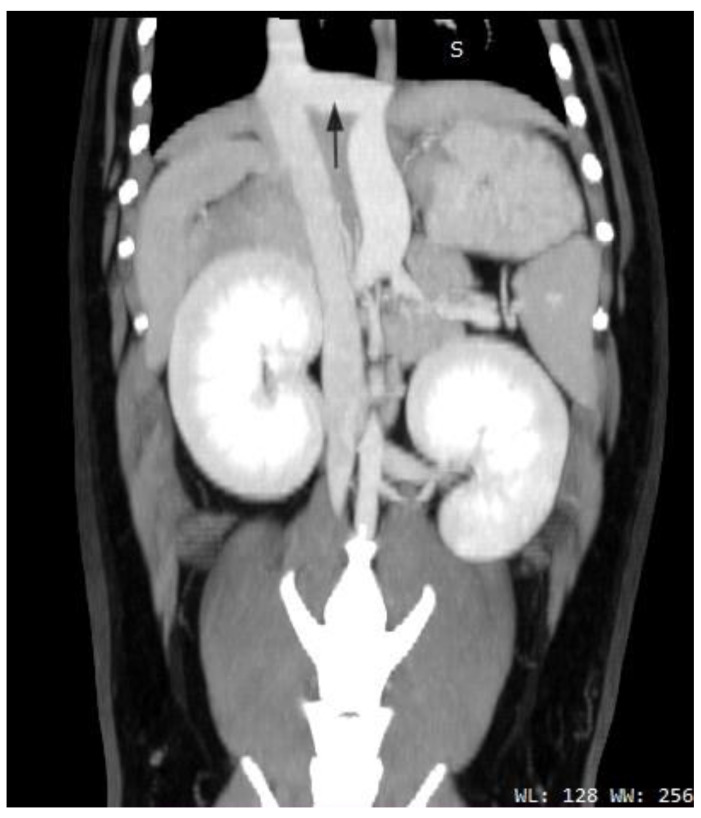
Coronal contrast-enhanced computed tomography image of microhepatia and an extrahepatic portosystemic shunt of the spleno-gastro-phrenic type (arrow) in a 1-year-and-7-month-old mixed breed female dog.

Cranial to the left kidney, an anomalous vessel with high blood flow velocity (80 cm s^−1^) was identified. The contrast-enhanced computed tomography showed an extrahepatic portosystemic shunt of the spleno-gastro-phrenic type with severe portal vein atresia (Figure 6). Although cellophane banding was performed, the clinical signs did not completely resolve.

### 2.5. Case E (Cat)

**Figure 7:**
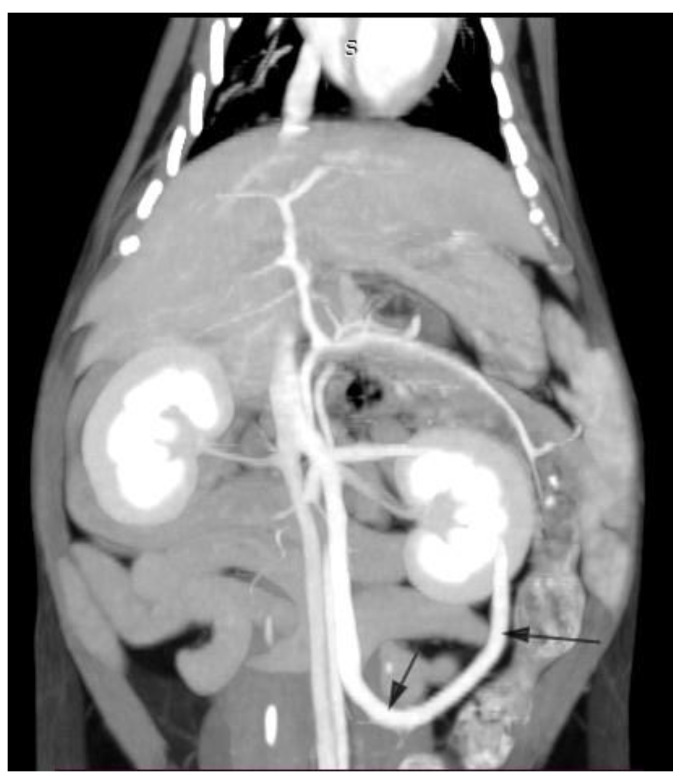
Coronal contrast-enhanced computed tomography image of an extrahepatic portosystemic shunt arising from the left colic vein (arrows) in a 6-month-old domestic short-haired male cat.

A domestic short-haired 6-month-old male cat presented with a history of at least two epileptoid seizures. The cat had copper-coloured eyes and elevated serum bile acids. This signalment and presentation is typical of portosystemic shunts in cats [12]. Contrast-enhanced computed tomography showed two branches of the caudal vena cava that joined at the level of the renal veins (Figure 7). In addition, an extrahepatic portosystemic shunt was found, arising from the left colic vein and taking a caudal loop-like course, joining the left branch of the caudal vena cava. This is a common finding in left colic shunts [13]. The liver volume was within normal limits, which is also a common finding in cats with portosystemic shunts [10]. The shunting vessel was surgically closed with cellophane banding.

## 3. Anatomy of the Portal Venous System

In humans, the inferior mesenteric vein drains into the splenic vein, which merges with the superior mesenteric vein to form the portal vein [1]. In dogs and cats, the portal vein is formed by the convergence of the cranial and caudal mesenteric veins. From the left side, the splenic vein, which accompanies the corresponding artery and receives the left gastric vein, contributes to portal vein formation [14]. From the right side, the gastroduodenal vein, the right gastric vein, and the gastroepiploic veins merge with the portal vein before entering the hepatic parenchyma. In humans and dogs, the portal vein divides into a right and a left branch. In cats, however, the portal vein divides into a right, a central, and a left portal branch, which supply the hepatic lobes with intrahepatic subdivisions [1,14].

## 4. Congenital Portosystemic Shunts

### 4.1. Embryology of the Portal Venous System

The embryonic development of the portal and systemic venous systems in humans, dogs, and cats is comparatively similar. The systemic venous system develops from the anterior and posterior cardinal veins, whereas the portal venous system develops from the left and right vitelline veins. The development of the portal venous system is complex. The vitelline veins develop on the anterior surface of the yolk sac, cross the septum transversum, and open into the sinus venosus. Subsequently, the veins grow and connect through at least three communicating vessels (subhepatic-cranioventral duodenal, intermediate-dorsal duodenal, and caudal-ventral duodenal) to form the vitelline venous network around the duodenum [15]. The hepatic cords begin to grow into the intrahepatic portion of the vitelline veins, forming hepatic sinusoids. These parts selectively involute and form the intrahepatic portions of the portal vein and hepatic veins. The extrahepatic left vitelline vein disappears, whereas the right one develops into the inferior (caudal) vena cava, the extrahepatic part of the portal vein, and the superior mesenteric vein [16,17].

Based on their anatomical location, congenital portosystemic shunts are divided into extrahepatic and intrahepatic shunts.

### 4.2. Congenital Extrahepatic Portosystemic Shunts

Extrahepatic shunts can be classified in humans, dogs, and cats following the same functional classification proposed by Morgan and Superina [6]. Type 1 or end-to-side extrahepatic portosystemic shunts connect the portal and systemic veins directly, with no significant portal vein flow to the liver. They develop due to excessive involution of the vitelline venous network around the duodenum and the resulting confluence of the portal vein directly with the inferior (caudal) vena cava. Type 2 or side-to-side extrahepatic portosystemic shunts have a connection between the portal and systemic venous systems (i.e., vena cava, left phrenic vein, azygos vein, renal veins, internal thoracic, or pelvic veins) [6,18]. These connections most commonly emerge from the left gastric vein, the left colic vein, the splenic vein, the mesenteric veins, and the superior rectal vein. Cranial to the shunt, the portal vein is usually hypoplastic [19,20]. These shunts develop due to the persistence of the right vitelline vein or due to the persistence of communications between the subcardinal and vitelline veins during the development of the inferior (caudal) vena cava. Extrahepatic portosystemic shunts are usually single; however, double shunts have also been reported [21]. The extrahepatic portosystemic shunts are named based on the name of the portal vessel from which they originate and the name of the first systemic vein into which they drain, e.g., a left gastrocaval shunt [22].

### 4.3. Congenital Intrahepatic Portosystemic Shunts

The intrahepatic portosystemic shunts are communications between the portal vein and hepatic or perihepatic veins with a diameter greater than 1 mm. Based on location and the number of communications, the shunts are divided into four types. Type 1 represents communication between a single vessel in the central part of the liver and a main branch of the portal vein and the inferior (caudal) vena cava. It develops due to persistence of the right vitelline vein. Type 2 represents single vessel communication in the peripheral parts of the liver in one segment. Type 3 represents communication through an aneurysm. Type 4 represents multiple small communications across the liver lobes. Intrahepatic shunts types 2–4 develop due to persistence of the communications between the vitelline venules [23]. The described classification is mostly used in humans due to the greater availability of detailed imaging modalities used in regular clinical practice; however, with the implementation of advanced imaging modalities in veterinary medicine, it is also increasingly used in cats and dogs. The intrahepatic portosystemic shunts in cats and dogs are traditionally divided into left-, right-, and central-divisional, based on the side of inflow into the inferior (caudal) vena cava [5].

Patent ductus venosus is known as a type 5 intrahepatic portosystemic shunt. During embryonic development, the umbilical vein is connected to the inferior (caudal) vena cava by the ductus venosus. Normally, it spontaneously closes soon after birth; however, in some occasions it remains patent. A patent ductus venosus acts like an intrahepatic shunt, which can lead to portal vein hypoplasia [24,25].

### 4.4. Aetiology of Congenital Portosystemic Shunts

In humans, congenital portosystemic shunts are very rare and are associated with numerous cardiovascular, hepatobiliary, urogenital and gastrointestinal malformations, and several genetic syndromes. Most of these anomalies are associated with extrahepatic shunts, whereas anomalies associated with intrahepatic shunts are not so prevalent [26]. With the advancements of radiologic imaging in the last decade, intrahepatic congenital portosystemic shunts are being increasingly recognized, especially in infants or prenatally. It is now known that these types of shunts do not need to be treated, as most will close spontaneously by the age of one year [27,28]. In dogs, congenital portosystemic shunts show a predilection for certain purebreds, with equal incidence in males and females. In addition, in the same breed, only extra- or intrahepatic congenital portosystemic shunts are usually seen; extrahepatic shunts are usually seen in smaller and toy breeds, and intrahepatic shunts are usually seen in larger dog breeds. Furthermore, the position of the intrahepatic portosystemic shunt is associated with the country of origin [9]. In cats, domestic shorthairs are most often affected; however, several purebred cats are predisposed to developing congenital portosystemic shunts. It has also been noted that male cats are more commonly affected than female cats [12]. All the above indicates a genetic basis of the disease. The mode of inheritance of extrahepatic portosystemic shunts is most likely polygenic, with multiple genomic regions being highly associated with the disease, whereas many intrahepatic shunts show autosomal recessive or digenic modes of transmission [9]. A strong candidate pathway involved in the development of intrahepatic portosystemic shunts is the aryl hydrocarbon receptor (AHR) pathway, which causes the transcription of several cytochrome P-450 genes and is necessary for the developmental closure of the ductus venosus [29,30].

## 5. Acquired Portosystemic Shunts

Acquired portosystemic shunts are portocaval pathways that develop in response to portal hypertension, where hemodynamic, angiogenic, and anatomic factors lead to the opening of pre-existing collaterals between the portal and systemic venous system [31]. More than twenty different portosystemic shunt pathways have been reported. The most common are collaterals between the esophageal branch of the left gastric vein and the esophageal branches of the azygos vein, the superior (cranial) rectal vein and the middle/lower (caudal) rectal veins, the paraumbilical veins and the superior (cranial) epigastric veins, the splenic vein and the renal/suprarenal/gonadal vein, and the colic veins and the retroperitoneal veins [31,32]. Longstanding increased flow through the collaterals causes the dilation of veins and the formation of varices, which can be classified based on their anatomical location into gastric, oesophageal, omental, gallbladder, abdominal wall (*caput medusae*), duodenal, colic, rectal, and retroperitoneal varices [32].

Based on anatomical location, the aetiologies of portal hypertension are divided into prehepatic, hepatic, and posthepatic. The most common prehepatic causes are thrombosis of the portal vein or splenic vein and arteriovenous fistulae. The most common hepatic causes are cirrhosis, hepatitis, chronic pancreatitis, and drug toxicity. The most common posthepatic causes are obstruction of the inferior (caudal) vena cava, right-sided heart failure, dirofilariasis, and hepatic vein thrombosis. Portal hypertension can also be secondary to portal vein hypoplasia or atresia [33,34]. Acquired portosystemic shunts and portal hypertension can also develop after the treatment of congenital portosystemic shunts, depending on the degree of portal vein hypoplasia [35]. Portal hypertension is more common in dogs than in cats because some breeds are predisposed to certain forms of hepatopathies [3].

## 6. Diagnostic Imaging of Portosystemic Shunts

Given its capacity to accurately evaluate abdominal structures and major vessels and their flow dynamics without the need of anaesthesia, ultrasonography has become the imaging technique of choice for the diagnostic work up of portosystemic shunts in humans and in small animals. In humans, ultrasonography is usually followed by contrast-enhanced computed tomography in order to better define the exact shunt anatomy [2]. In dogs and cats, ultrasonography is usually the first diagnostic imaging procedure employed, and it is increasingly being followed by contrast-enhanced computed tomography. The reported true positive rates (sensitivity) of ultrasonography were 74% and 50–100% for humans and dogs and cats, respectively, whereas the specificity in both animals was 100% [36,37,38].

A 10-step systematic approach for the evaluation of portosystemic shunts in dogs and cats was suggested by d’Anjou [10]. Animals should be fully shaved ventrally in order to gain good visibility of the whole abdomen, diaphragm, and major vessels in the thorax. A linear or microconvex transducer should be used to examine the animals in subcostal, lateral, and dorsal recumbency, and a right-sided intercostal approach should be used to meticulously visualize all the abdominal structures [10,39].

### 6.1. Signs of Portal Hypertension

Portal hypertension is associated with acquired portosystemic shunts, whereas it is usually not associated with congenital portosystemic shunts [39,40]. Most humans, dogs, and cats with portal hypertension exhibit one or more ultrasonographic sings of portal hypertension: overt ascites, a dilated portal vein, biphasic or reverse blood flow in the portal vein, splenomegaly, recanalization of the umbilical vein, portosystemic varices, and oedema of the abdominal organs (the gallbladder wall, gastrointestinal tract, and pancreas, which has a characteristic “tiger-stripe” appearance) [39,41,42].

### 6.2. Liver

In congenital portosystemic shunts, there is a reduction of portal venous flow to the liver and a consequent reduction of the inflow of hepatotropic factors that causes hepatic atrophy and results in a smaller liver with uniform and normal echogenicity [40,43,44]. This is most evident in dogs, whereas it is less pronounced in cats [10]. In humans, congenital portosystemic shunts are strongly associated with benign and malignant liver tumours. Therefore, regular imaging follow-up of these patients is required. In acquired portosystemic shunts, liver size varies according to the cause of portal hypertension. In liver cirrhosis and in chronic hepatitis, the liver is usually decreased in size, whereas in acute hepatitis and diffuse neoplasia, the liver is usually enlarged. These changes are accompanied by changes in echogenicity and nodular formation [10,39,41]. In humans, liver size is estimated by liver length in the midclavicular line, which normally increases from 4.5 cm at birth to 12.5 cm in adulthood [45]. In dogs and cats, the size of the liver is estimated by subjective assessment using indirect criteria, such as shape, caudal margin extension, contour, position of the spleen, and the relative size of the gallbladder. However, when evaluating the liver dimensions of a person or an animal, the age, size, and constitution of the individual should also be taken into account [44,46].

### 6.3. Intrahepatic Portal Veins

Hepatic veins and intrahepatic portal veins can be ultrasonographically differentiated based on vessel wall hyperechogenicity. In contrast to hepatic veins, the portal vein walls are hyperechoic regardless of the angle of the probe and the direction of blood flow [47]. Normally, portal veins are distributed homogenously in all the hepatic lobes and their diameter gradually decreases toward the periphery. In many portosystemic shunts, hepatic portal flow is decreased, resulting in small portal veins. However, there are no validated criteria to assess the size of the intrahepatic veins, so this assessment is only subjective [10,39,44,46].

Detailed visualisation of the intrahepatic veins is necessary in order to evaluate the presence of intrahepatic portosystemic shunts. These can be seen as large, tubular, anechoic structures that connect to the portal and hepatic veins and subsequently drain into the inferior (caudal) vena cava [48]. In dogs and cats, intrahepatic portosystemic shunts can be divided into left-divisional, right-divisional, and central-divisional intrahepatic shunts based on their direction towards the anomalous vascular curves. The left divisional shunt usually dilates and forms an ampulla before opening into the inferior (caudal) vena cava. Sometimes the intrahepatic portal vein runs close to the inferior (caudal) vena cava and connects with it via a foramen (a window-type intrahepatic shunt) [5,10,39,49]. It has been proposed that such a classification should be replaced by a more elaborate classification that is also used in humans (*vide supra*), which requires additional imaging diagnostics with computed tomography and magnetic resonance imaging in order to describe the complex intrahepatic connections between the hepatic and portal veins [3].

On Doppler ultrasonography, antegrade flow is seen in the intrahepatic portosystemic shunt vessels. The afferent feeding (portal) vessel of the shunt usually shows increased flow velocity with phasic waveforms due to transmitted pulsations of the heart, whereas the draining vessel shows continuous flow with a flattened waveform due to increased inflow instead of the usual triphasic spectral waveform of the hepatic veins. If the shunt vessels form a focal varix, it is seen as a round cyst-like structure with turbulent flow [50]. Moreover, in contrast-enhanced ultrasounds, the shunt draining the hepatic veins is enhanced earlier than the other hepatic veins [51].

### 6.4. Portal Vein Size

The portal vein reaches its largest diameter at the transverse fissure of the liver. There, it approaches the size of the aorta and the inferior (caudal) vena cava. In humans, the portal vein diameter increases from 4.5 mm at birth to 11 mm in adulthood, and does not increase above 16 mm [52,53]. In dogs and cats, the portal vein diameter varies significantly. Therefore, the ratio comparing the maximal portal vein diameter and the maximal diameter of the aorta (at systole) is assessed. The normal portal-vein-to-aorta diameter ratio in dogs and cats is 0.7–1.25 [10,39]. In congenital intrahepatic portosystemic shunts and acquired portosystemic shunts due to chronic liver diseases, the portal vein is enlarged or is normal in size [54]. In contrast, in congenital extrahepatic portosystemic shunts, the diameter of the portal vein is significantly decreased cranial to the beginning of the shunt, especially cranial to the inflow of the gastroduodenal vein, because blood flow is redirected to the systemic circulation [10,39,40,54]. Sometimes, the hypoplastic portal vein is very difficult to identify because it measures only a few millimetres, is displaced more cranially, and can be mistaken for a portal vein tributary, a portosystemic shunt, or a hepatic artery. If the portal-vein-to-aorta ratio is larger than 0.8 cranially to the gastroduodenal vein, an extrahepatic portosystemic shunt can be excluded [10,39].

### 6.5. Portal Vein Blood Flow

Portal vein blood flow can be evaluated using Doppler techniques. The portal vein should be examined longitudinally from the subcostal approach, and the mean and maximal velocity should be measured [55,56]. A normal portal vein Doppler trace should be hepatopetal (towards the liver) and monophasic with little variation in blood flow velocity. The normal mean velocity of portal vein flow ranges from 0.11 to 0.22 m s^−1^ in humans, 0.15 to 0.20 m s^−1^ in dogs, and 0.10 to 0.18 m s^−1^ in cats [37,39,57]. Care should be taken to use appropriately large sample volumes and to not exceed the angle of 60° between the portal vein and the transducer.

In intrahepatic portosystemic shunts, the mean velocity of portal vein flow is increased (>0.50 m s^−1^), whereas in extrahepatic portosystemic shunts, the mean velocity of portal vein flow is decreased (<0.15 m s^−1^) or reversed [10,39]. In the case of portal hypertension and acquired portosystemic shunts, portal flow is usually reduced (<0.15 m s^−1^), hepatofugal (reversed), or bidirectional (changing directions with the breathing cycle) [10,40,57,58]. A normal, gently undulating portal vein waveform could be influenced by the systemic circulation with variable pressure, which may influence the waveform, producing irregular blood flow spectra.

### 6.6. Portal Vein Tributaries

The main portal vein tributaries should be followed from the periphery to the portal vein, with the probe directed cranially, and the blood flow direction should be investigated using colour Doppler. Blood flow is normally directed toward the portal vein and the liver. Any vein of the portal system with blood flowing away from the liver towards the periphery should raise suspicion of an extrahepatic portosystemic shunt [10]. The shunts can originate from the portal vein or any tributary; however, ultrasonographic detection of the true origin of the shunt is usually difficult. Prior to surgery, the most important step is to evaluate the number of shunts, the exact site of their termination, and their size. Extrahepatic shunts usually terminate in the inferior (caudal) vena cava superior to the right renal artery or in the azygos vein [10,39,40,56].

A shunt originating from the left gastric vein often follows a tortuous course, terminating into the left phrenic vein, the inferior (caudal) vena cava, or the azygos vein. With an ultrasound, it may be visualized as a large calibre vessel coursing from a cranial direction to join the gastrosplenic vein close to its termination in the main portal vein [59].

A left colic shunt arises from the left colic vein, often taking a caudal course prior to making a straight angle turn to join the caval circulation via either the inferior (caudal) vena cava or the common iliac vein. With an ultrasound, it is recognized as a large calibre vessel with hepatofugal flow into a distended caudal mesenteric vein [13].

A splenocaval shunt originates from the splenic vein as an aberrant left gastric vein, terminating at the plane of the epiploic foramen into the prehepatic part of the inferior (caudal) vena cava [60].

### 6.7. Inferior (Caudal) Vena Cava

The vena cava is usually enlarged cranially to termination of the extrahepatic or intrahepatic portosystemic shunt, and blood flow at the site of shunt termination shows turbulent flow on colour Doppler. This is a very useful sign that points toward the location at which the shunt terminates [10,39,43,54,56]. Additionally, the vena cava should also be inspected for possible concurrent anomalies, such as segmental atresia and double vena cava [10,40].

### 6.8. Azygos Vein

Normally, the azygos vein cannot be seen ultrasonographically. However, when a portoazygous shunt is present, the azygos vein becomes prominent as a vessel coursing right to the descending aorta, with blood flow directed cranially [10]. The most common subtype of portoazygous shunts is the left gastroazygous shunt, which arises from the left gastric vein and drains into the azygos vein [8].

### 6.9. Acquired Portosystemic Shunts

Acquired portosystemic shunts are usually multiple and located at specific predisposed locations that can vary between subjects [10,61]. Acquired extrahepatic portosystemic shunts are seen as tubular hypoechoic structures that have hepatofugal flow on Doppler ultrasound [48]. Common examples of these shunts include oesophageal, paraoesophageal, and gastric varices that shunt blood toward the azygos vein; paraumbilical varices, which shunt blood towards the epigastric and external iliac veins; rectal and hemorrhoidal varices, which shunt blood towards the pudendal and internal iliac veins; and splenorenal varices (also known as a splenosystemic shunt), which shunt blood towards the left renal vein. The latter is identified as a longer vessel originating from the splenic vein and draining into the left renal vein with a complex pattern of short, tortuous vessels at the distal end. The ipsilateral gonadal vein is typically enlarged [10,40,48]. Splenorenal varices should be differentiated from the congenital extrahepatic splenocaval shunt, which, in contrast to splenorenal varices, course cranially [60].

When severe portal hypertension with enlarged portal vein tributaries with pulsatile, high speed blood flow, and multiple acquired portosystemic shunts are present, arterioportal shunts should be considered. Arterioportal shunts are not rare and can be congenital or acquired, resulting from cirrhosis, tumours, or trauma [48,62].

### 6.10. Kidneys

Due to the reduced hepatic metabolism of ammonium and the increased excretion of urate, ammonium urate kidney stones can be expected in subjects with congenital portosystemic shunts. They present as hyperechoic foci in the kidneys, renal pelvis, ureter, bladder, or urethra [11]. In addition, the kidneys can be enlarged due to increased renal blood flow, hormonal stimulation, or as a compensatory response to reduced liver function [63].

## 7. Conclusions

The present paper highlights the similarities and differences in portosystemic shunts between humans and small animals. Portosystemic shunts are very rare in humans and significantly more common in small animals. They can be congenital or acquired and are divided into intrahepatic and extrahepatic types. Congenital shunts are further divided into two types of intrahepatic shunts and five types of extrahepatic shunts. The etiology of congenital shunts is not yet fully understood, but it seems that the inheritance of extrahepatic shuts is most likely polygenic, whereas intrahepatic shunts show autosomal recessive or digenic modes of transmission with the aryl hydrocarbon receptor pathway often being involved. Acquired portosystemic shunts are portocaval pathways that develop in response to long standing portal hypertension. The diagnosis of portosystemic shunts is confirmed by ultrasonography, which is the first-choice imaging modality, by evaluating the signs of portal hypertension, the liver, intrahepatic portal veins, portal vein size, portal vein blood flow, portal vein tributaries, the inferior (caudal) vena cava, azygos veins, and the kidneys. This is usually followed by contrast-enhanced computed tomography in order to better visualise and define the exact type of shunt. Accordingly, it is important to understand the embryology, anatomy, pathology, and pathophysiology of portosystemic shunts to understand the findings of radiologic imaging and to direct appropriate treatment.

## Data Availability

The data supporting the findings of this study are private in order to protect personal data.
